# Primary Lung Tumors in Children: Insights from a Single-Center Case Series

**DOI:** 10.3390/jcm14072173

**Published:** 2025-03-22

**Authors:** Paola Borgia, Barbara Cafferata, Claudio Paratore, Lorenzo Anfigeno, Alessio Conte, Angelo Florio, Annalisa Gallizia, Marco Del Monte, Francesca Buffelli, Francesca Rizzo, Maria Beatrice Damasio, Pietro Salvati, Katia Perri, Alberto Garaventa, Teresa Battaglia, Virginia Livellara, Massimo Conte, Giovanni Arturo Rossi, Valerio Gaetano Vellone, Michele Torre, Carlo Castellani, Oliviero Sacco

**Affiliations:** 1 Pediatric Pulmonology and Respiratory Endoscopy Unit, IRCCS Istituto Giannina Gaslini, 16147 Genova, Italy; paolaborgia@gaslini.org (P.B.); angeloflorio@gaslini.org (A.F.); annalisagallizia@gaslini.org (A.G.); pietrosalvati@gaslini.org (P.S.); katiaperri@gaslini.org (K.P.); giovannirossi@gaslini.org (G.A.R.); carlocastellani@gaslini.org (C.C.); olivierosacco@gaslini.org (O.S.); 2 Pathology Unit, IRCCS Istituto Giannina Gaslini, 16147 Genoa, Italy; barbaracafferata@gaslini.org (B.C.); francescabuffelli@gaslini.org (F.B.); valeriovellone@gaslini.org (V.G.V.); 3 Department of Surgical Sciences and Integrated Diagnostic (DISC), University of Genoa, 16132 Genoa, Italy; claudio.paratore@virgilio.it (C.P.); micheletorre@gaslini.org (M.T.); 4 Pediatric Radiology Department, IRCCS Istituto Giannina Gaslini, 16147 Genoa, Italy; lorenzoanfigeno@gaslini.org (L.A.); francescarizzo@gaslini.org (F.R.); 5 Department of Neurosciences, Rehabilitation, Ophthalmology, Genetics, Maternal and Child Health, University of Genoa, 16126 Genoa, Italy; alessioconte.1995@gmail.com (A.C.);; 6 Clinical Oncology Unit, IRCCS Istituto Giannina Gaslini, 16147 Genoa, Italy; albertogaraventa@gaslini.org (A.G.); teresabattaglia@gaslini.org (T.B.); virginialivellara@gaslini.org (V.L.); massimoconte@gaslini.org (M.C.); 7 Pediatric Surgery, IRCCS Istituto Giannina Gaslini, 16147 Genoa, Italy

**Keywords:** lung tumor, pediatric oncology, inflammatory myofibroblastic tumor, carcinoid tumor, myoepithelial carcinoma, congenital peribronchial myofibroblastic tumor, pleuropulmonary blastoma

## Abstract

**Background**: Primary lung tumors in pediatric patients are rare, predominantly malignant, and present diagnostic challenges due to symptom overlap with more common conditions such as inflammatory processes or asthma. Evidence-based approaches for managing these rare neoplasms in childhood are scarce. This retrospective study reports the experience of a pediatric referral center in diagnosing and treating these tumors. **Methods:** Pediatric primary lung tumors treated at Giannina Gaslini Children’s Hospital between January 2016 and January 2024 were included. Data on clinical presentation, histopathology, imaging, treatment approaches, and outcomes were systematically collected and analyzed. **Results:** Nine patients (six males and three females) were identified, with a mean age (±SD) at diagnosis of 8.81 ± 5 years. The most common clinical manifestation was recurrent pneumonia (four patients), followed by persistent cough and wheezing (three patients). The average duration of symptoms before diagnosis was 12.8 months ± 12.2 months. Histopathological diagnoses were typical carcinoid tumors (*n* = 2), atypical carcinoid tumors (*n* = 2), inflammatory myofibroblastic tumors (*n* = 2), congenital peribronchial myofibroblastic tumor (*n* = 1), myoepithelial carcinoma (*n* = 1), and pleuropulmonary blastoma (*n* = 1). Radical surgery resulted in complete response for seven patients, with a median follow-up of 52 months (IQR 39 months). The myoepithelial carcinoma was treated with multimodal therapy, relapsed after 17 months, and adjuvant chemotherapy is currently ongoing. Neoadjuvant chemotherapy for the pleuropulmonary blastoma is currently ongoing. **Conclusions:** Primary lung tumors in children, though rare, may have favorable outcomes when appropriately managed. Nonspecific clinical presentations often contribute to diagnostic delays. This study highlights the critical need of thorough evaluation in cases of persistent, therapy-resistant aspecific respiratory symptoms. Early diagnosis, coupled with complete surgical resection, significantly improves prognosis.

## 1. Introduction

Primary lung tumors are extremely rare in pediatric patients, amounting to less than 0.2% of all malignancies in children, with an annual incidence of 0.049 per 100,000 infants [1]. Survival varies significantly by histology, with certain types like endocrine neoplasms and mucoepidermoid tumors showing 10-year survival rates of 92–100%, while others, such as small cell carcinoma, have a median survival of less than 5 months [2]. In neonates and infants, the most common tumor is pleuropulmonary blastoma, followed by infantile fibrosarcoma and lung interstitial tumors. In older children, inflammatory myofibroblastic tumors and carcinoid tumors are most commonly encountered [3,4,5]. The associated symptoms vary according to the type of tumor, its size, rate of growth, malignant potential, vascularity, and location [6]. Symptoms may include fever, cough, chest pain, hemoptysis, and respiratory distress. All of these are nonspecific and can be mistaken for manifestations of more common respiratory disorders like asthma and pneumonia, which may lead to diagnostic delays [7]. Imaging plays a crucial role in the diagnostic process, with chest X-rays frequently revealing persistent or recurrent pulmonary opacities that warrant further investigation. Computed tomography (CT) scans provide detailed information about localization, size, and possible lymph node involvement. If an endobronchial tumor is suspected, bronchoscopy is an essential tool for direct visualization and biopsy [8]. The gold standard for definitive diagnosis is histopathological and immunohistochemical analysis, with molecular and genetic studies, such as *DICER1* mutation screening in pleuropulmonary blastoma, increasingly used to refine diagnosis [4]. Treatment strategies vary according to histology and disease extent. Complete surgical resection is the treatment of choice for localized tumors, while chemotherapy and radiotherapy are typically reserved for unresectable or metastatic disease [9]. Given the rarity of primary lung tumors in pediatric patients and the limited data available in the literature, this study aims to provide further insight by reporting nine cases diagnosed and followed at the Giannina Gaslini Children’s Hospital, offering additional details into the clinical, radiologic, and histopathologic characteristics, as well as the management and prognosis of these rare neoplasms.

## 2. Materials and Methods

### 2.1. Patients

The study recruited nine children and adolescents diagnosed with primary lung tumors at Giannina Gaslini Children’s Hospital from 1 January 2016, to 1 January 2024. Inclusion criteria were (i) age under 18 years; (ii) histopathological diagnosis of a primary lung tumor, based on clinical, radiologic, and histopathologic evaluations. Exclusion criteria were (i) secondary lung tumors or metastases; (ii) incomplete medical records. Cases were identified by reviewing the hospital’s electronic medical records and the pathology database. The search was conducted using International Classification of Diseases (ICD) codes: ICD 162 for malignant tumors of the trachea, bronchi, and lungs, and ICD 212 for benign tumors in the same regions. Any histological subtype of primary lung tumors was included.

### 2.2. Ethical Aspects

This study was approved by the Institutional Review Board (IRB) of Giannina Gaslini Children’s Hospital (protocol code 02/25). Consent for participation was obtained from the caregivers in accordance with ethical standards for research involving human participants.

### 2.3. Study Outcomes

The primary outcome of this study was to characterize clinical presentation, diagnostic approach, and treatment outcomes of pediatric patients with primary lung tumors. Secondary outcomes included data on the distribution of histological tumor types, clinical progression, and follow-up results. Additional analyses assessed the duration of symptoms before diagnosis, the rate of complete resection, and the incidence of postoperative complications.

### 2.4. Study Design

This is a single-center, retrospective cohort study. Data were collected from electronic medical records. Demographic information, including age at diagnosis and gender, was recorded. Clinical presentation was assessed based on symptoms and their duration before diagnosis. Diagnostic procedures included imaging findings, biopsy results, and histopathological classification. Tumors were classified based on histopathological features observed in the biopsy samples. Immunohistochemical analyses and molecular data, including *DICER1* mutation screening, were also reviewed. The treatment modalities considered included surgical resection, chemotherapy (neoadjuvant or adjuvant), and radiotherapy. Clinical outcomes were evaluated in terms of treatment response, follow-up duration, and the overall postoperative course.

### 2.5. Statistical Analysis

Descriptive statistics for the entire cohort are reported. Continuous variables are presented as the mean ± standard deviation (SD) or median with interquartile range (IQR), depending on the results of normality testing. The normality of the data distribution was assessed using the Shapiro–Wilk test. Age at diagnosis and duration of symptoms followed a normal distribution and are therefore presented as mean ± SD. Follow-up time did not follow a normal distribution and is therefore presented as median ± IQR. Categorical variables, such as gender and histopathological diagnosis, are presented as frequencies and percentages. The median follow-up time was calculated for patients with complete follow-up data. All statistical analyses were conducted using the Statistical Package for the Social Sciences for Windows v. 14 (IBM Inc., Chicago, IL, USA).

## 3. Results

Clinical data of the patients are shown in Table 1. The mean age at diagnosis was 8.81 years (range 16 months to 16 years) with a male predominance (*n* = 6). Only one patient was asymptomatic at diagnosis and incidentally discovered by chest CT scan following a chest trauma. The most common presenting symptom was recurrent pneumonia in four patients. The mean duration of symptoms was 12.8 ± 12.2 months. Pathologic diagnoses included carcinoid tumors (typical *n* = 2; atypical *n* = 2), inflammatory myofibroblastic tumors (*n* = 2), congenital peribronchial myofibroblastic tumor (*n* = 1), myoepithelial carcinoma (*n* = 1), and pleuropulmonary blastoma (*n* = 1). Endobronchial biopsy was performed in five cases before surgery. Two patients underwent segmentectomy, while the remaining five underwent lobectomy of one (*n* = 2) or two lobes (*n* = 3). Only one child with atypical carcinoid later required pneumonectomy because of hilar lymph node involvement. All seven patients showed complete response after radical surgery, with a median follow-up of 52 months (IQR 39 months). The patient with myoepithelial carcinoma was treated with multimodal therapy (neoadjuvant chemotherapy, surgical tumor debulking, adjuvant chemotherapy, and locoregional radiotherapy), developed disease recurrence 17 months after completing treatment, and is currently undergoing adjuvant chemotherapy. The patient with pleuropulmonary blastoma is currently receiving neoadjuvant chemotherapy as part of the treatment regimen. The postoperative course of all surgical patients was uneventful.

### 3.1. Carcinoid Tumors

**Figure 1:**
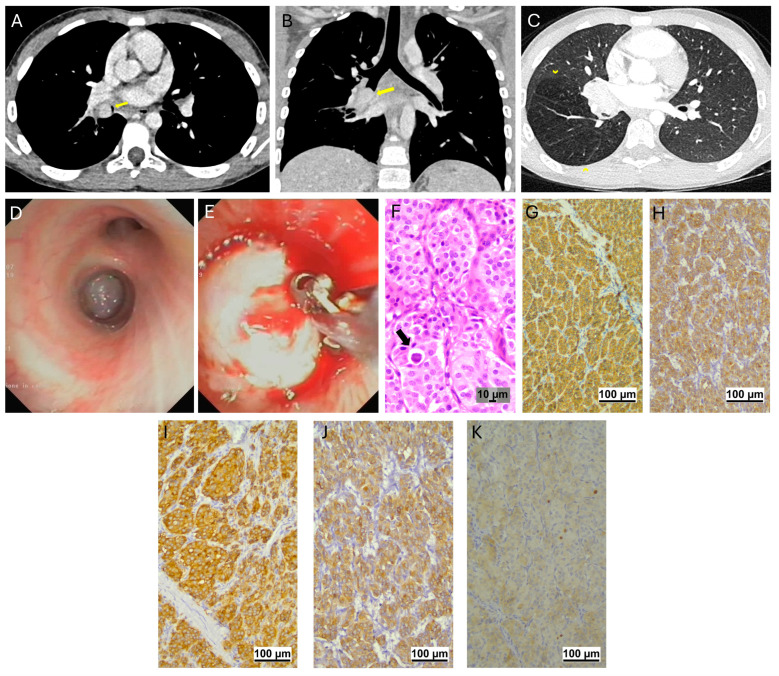
Typical carcinoid tumor (patient 2). Volumetric chest CT scan with post-contrast material (CM) acquisition, 0.6 mm slice thickness, and 1 mm multiplanar reconstruction (MPR). Axial (**A**) and coronal (**B**) reconstructions show a contrast-enhanced solid endobronchial tumor within the right intermediate bronchus (arrow). Parenchymal air trapping below the lesion involving the right inferior lobe is noted (arrowheads in (**C**)). Endoscopic appearance. Note the whitish endobronchial neoformation and its hypervascular nature (**D**), as indicated by the tendency for bleeding after biopsy (**E**). Histopathologic features. Hematoxylin and Eosin: groups of neoplastic cells with an eosinophilic body intermixed (arrow in (**F**)). On IHC (immunohistochemistry), strong cytoplasmic positivity for CD56 (**G**), cytokeratins (**H**), synapthophysin (**I**), and chromogranin (**J**). Ki67 is <1% 20× (**K**).

**Figure 2:**
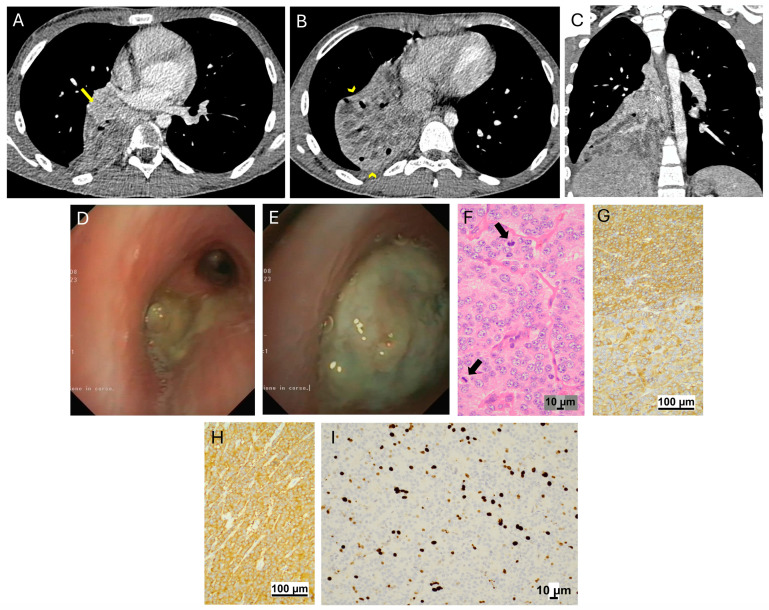
Atypical carcinoid tumor (patient 3). Volumetric chest CT scan with post-CM acquisition, 0.6 mm slice thickness, and 1 mm MPR. Axial scans show a solid endobronchial mass with contrast enhancement located in the right intermediate bronchus (arrow in (**A**)). Atelectatic areas with cystic bronchial dilatation are evident (arrowheads in (**B**)). Coronal reconstruction (**C**) confirms the obstruction and atelectatic parenchyma within the lower lobe with cystic dilatation of bronchi. Extra bronchial tissue extension is also visible (*). Endoscopic features. Yellowish mass covered with purulent secretions completely obstructing the lumen of the intermediate bronchus (**D**). Note the irregular surface of the mass after aspiration (**E**). Histopathologic features. Hematoxylin and Eosin: the tumor has a solid growth pattern of cells showing atypia with easily identifiable mitoses (arrow in (**F**)). On IHC, strong cytoplasmic positivity for Chromogranin (**G**) and Synaptophysin (**H**) is evident. Ki67 index is 10% (**I**).

Four male patients, aged between ten and sixteen years, were followed for carcinoid tumors (typical *n* = 2; atypical *n* = 2). Three cases had a medical history of recurrent pneumonia, the other of persistent cough and wheezing. There was a latency between the first symptoms and the final diagnosis of six months to three years. At CT scan, three cases had involvement of the intermediate bronchus, one of the right lower bronchus. In all, a preoperative histological diagnosis followed the flexible bronchoscopy (Figure 1 and Figure 2). Three patients had low-grade tumors and primary surgery resulted in complete resection without any microscopic residual. The fourth one (patient 3) had a high-grade atypical lesion with lymph node involvement and following the first intervention had a pneumonectomy and radical lymphadenectomy for an atypical carcinoid tumor with lymph node metastasis. Postoperative surveillance consisted of clinical visits with a chest CT every six months during the first two years, followed by an annual chest CT for at least five years, and biennial imaging thereafter for a period of up to ten years. All patients achieved complete remission, and their mean follow-up duration was 51 months (range: 12 to 84 months).

### 3.2. Inflammatory Myofibroblastic Tumors

**Figure 3:**
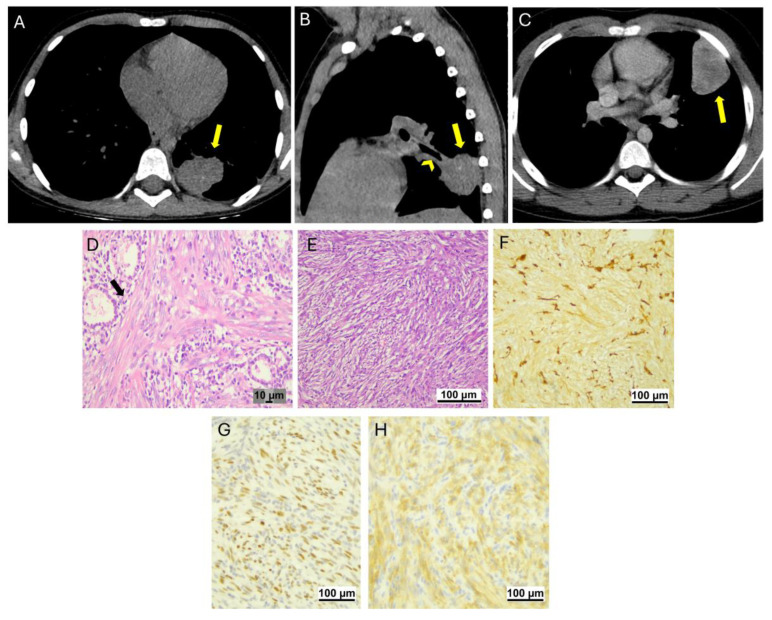
Inflammatory myofibroblastic tumors. Volumetric chest CT scan with post-CM acquisition, 0.6 mm slice thickness, and 1 mm MPR. Axial scan with mediastinal window ((**A**), patient 5) reveals a bilobed round lesion in the left lower lobe (arrow), with substantially homogeneous low-contrast enhancement. Sagittal scan with mediastinal window ((**B**), patient 5) demonstrates the lesion (arrow) situated posteriorly to the terminal segment of the posterior bronchial branch for the left lower lobe (arrowhead). Axial scan with mediastinal window ((**C**), patient 6) shows a polylobed round lesion in the left upper lobe (arrow), adjacent to the pleura, with heterogeneous contrast enhancement. Histopathologic features. The Hematoxylin and Eosin stain reveals a proliferation of spindle cells (arrow in (**D**)) mixed with normal lung parenchyma (patient 5). Hematoxylin and Eosin: proliferation composed of compact fascicular spindle cells within the collagenous stroma ((**E**), patient 6). IHC shows characteristic cytoplasmic positivity for smooth muscle actin ((**F**), patient 6) and nuclear positivity for p53 ((**G**), patient 6). ALK immunohistochemistry shows cytoplasmic positivity ((**H**), patient 6).

Two male patients, aged 9 and 11, were diagnosed with inflammatory myofibroblastic tumors (IMTs). One of them (patient 5) had been experiencing recurrent chest pain for six months before being diagnosed, while the other (patient 6) was diagnosed incidentally by a chest CT for trauma-induced chest pain. The histological diagnosis was confirmed through thoracoscopic biopsy for one lesion located in the left lower lobe, while for the second case, located in the lingula, the definitive histological diagnosis was confirmed post-surgery (Figure 3). There was no evidence of lymph node involvement or distant metastases in either case. Both patients underwent atypical lobectomy and remained disease-free after 5 and 8 years, respectively. The follow-up included clinical evaluation and an annual chest CT for the first two years, followed by a biannual chest CT for at least 11 years.

### 3.3. Congenital Peribronchial Myofibroblastic Tumor

**Figure 4:**
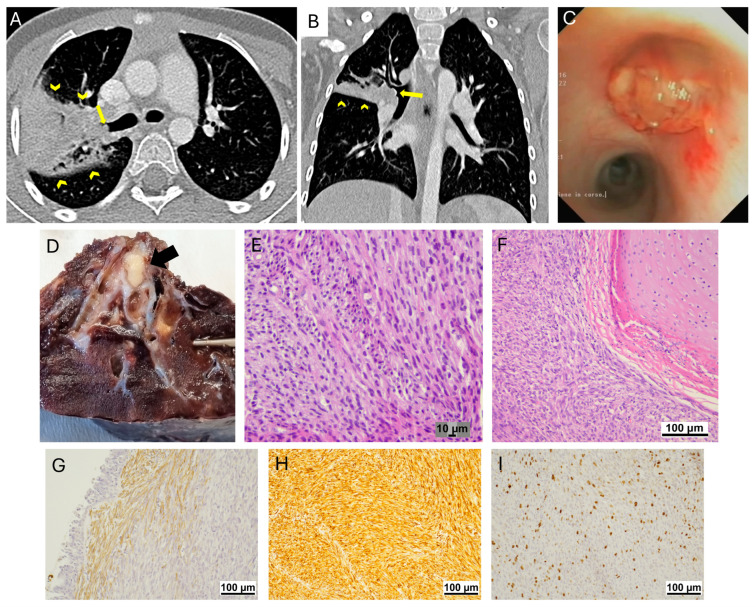
Congenital peribronchial myofibroblastic tumor (patient 7). Volumetric chest CT scan with post-CM acquisition, 0.6 mm slice thickness, and 1 mm MPR. Axial (**A**) and coronal (**B**) reconstructions show an endobronchial lesion (arrow) obstructing the right upper bronchus, resulting in post-obstructive atelectasis of the apical and posterior segments of the right upper lobe (arrowheads). Bronchoscopic examination. A solid lesion with a pink papillary surface obstructing the orifice of the right upper bronchus (**C**). Histopathologic features. An endobronchial lesion was detected on gross examination. The lesion has a typical yellow color (arrow in (**D**)). The tumor is composed of fascicles of spindle cells and no necrosis is present (**E**,**F**). IHC: AML is focally positive (**G**) and Vimentin is positive (**H**). Ki67 index is 10% (**I**).

A 5-year-old girl (patient 7) with no significant past medical history presented with a 1-year history of recurrent right upper lobe pneumonia. A CT scan revealed an endobronchial lesion within the right upper bronchus (Figure 4). No suspicious pulmonary nodules or extrathoracic lesions were observed. Tissue biopsies obtained through flexible bronchoscopy revealed small spindle-shaped cells with low mitotic activity, arranged in fasciculate formations and vascularized by a thin network of pre- and post-capillary vessels lacking well-defined walls, with no evidence of necrosis or hemorrhage. Immunohistochemical studies demonstrated a strong positive reaction for vimentin (Figure 4). These findings were consistent with a diagnosis of congenital peribronchial myofibroblastic tumor. A right upper lobectomy was performed, and no lymph node metastases were detected. Surveillance visits with chest CT have shown no evidence of recurrent disease six months after lung surgery.

### 3.4. Myoepithelial Carcinoma

**Figure 5:**
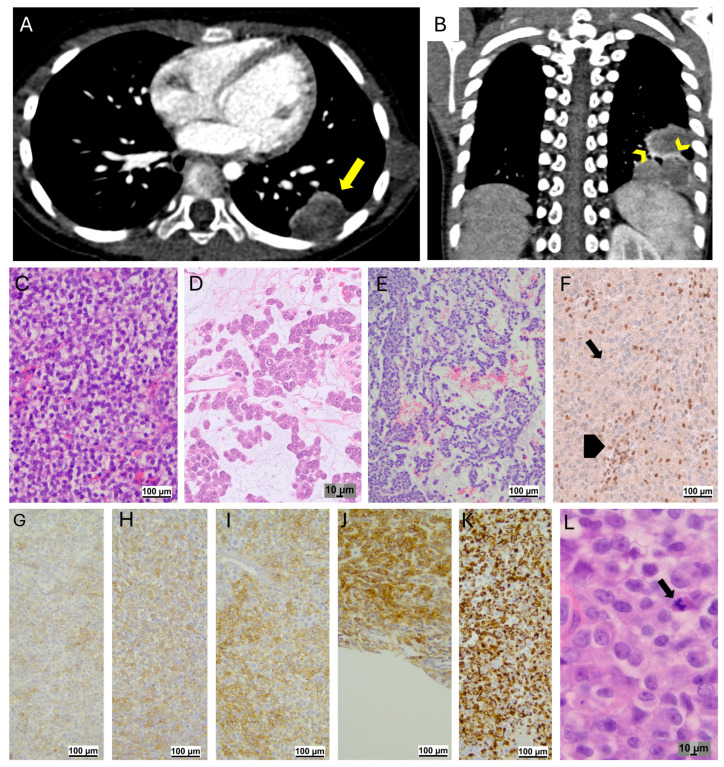
Myoepithelial carcinoma (patient 8). Volumetric chest CT scan with post-CM acquisition, 0.6 mm slice thickness, and 1 mm MPR. Axial scan with mediastinal window shows a polylobed round lesion in the left lower lobe (arrow in (**A**)), adjacent to the pleura. Coronal scan: the lesion has heterogeneous contrast enhancement with a hyperdense halo (arrowhead in (**B**)). Histopathologic features. Hematoxylin and Eosin: the tumor is composed of a predominant small round blue cell component (**C**) and focal areas with rhabdoid features (**D**). A focal myxoid pattern is also detected (**E**). Immunohistochemistry for INI-1 is negative (arrow: negative stain detected in tumor cells; arrowhead: positive internal control (**F**)). CD99 (**G**), SMA (**H**), EMA (**I**), CD117 (**J**), vimentin (**K**) stains are positive. Mitoses were easily identified (arrow in (**L**)).

A 16-month-old girl (patient 8) with persistent cough and dyspnea for the past 3 months was diagnosed in another institution with pleuropulmonary blastoma in the anterior mediastinum and left pulmonary apex. She underwent non-radical surgery and was treated with ifosfamide and doxorubicin. At the age of 5, she was referred to our hospital for further evaluation and management because of persistent fever, cough, and dyspnea. A CT scan showed a polylobed round lesion exhibiting heterogeneous contrast enhancement with a hyperdense halo (Figure 5). A subsequent thoracoscopic biopsy confirmed the diagnosis of myoepithelial carcinoma upon pathological analysis (Figure 5). The tumor exhibited an immunohistochemical profile characterized by a complete loss of INI1 (SMARCB1) expression, coupled with epithelial membrane antigen (EMA), S100, p63, and CK MNF116 positivity. Genetic analysis showed a compound somatic heterozygous mutation in the *DICER1* gene (c.5566G>A and c.4390_4391dup) which is not included in the Catalogue of Somatic Mutations in Cancer (COSMIC) [9]. The patient received various chemotherapy regimens including vincristine, irinotecan, temozolomide, cisplatin, carboplatin, and etoposide, which consistently resulted in no tumor shrinkage. She underwent surgical intervention including debulking and subsequently locoregional radiotherapy. Seventeen months later, local relapses were detected, and in order to limit tumor development, chemotherapy with vinorelbine-cyclophosphamide was planned.

### 3.5. Pleuropulmonary Blastoma

**Figure 6:**
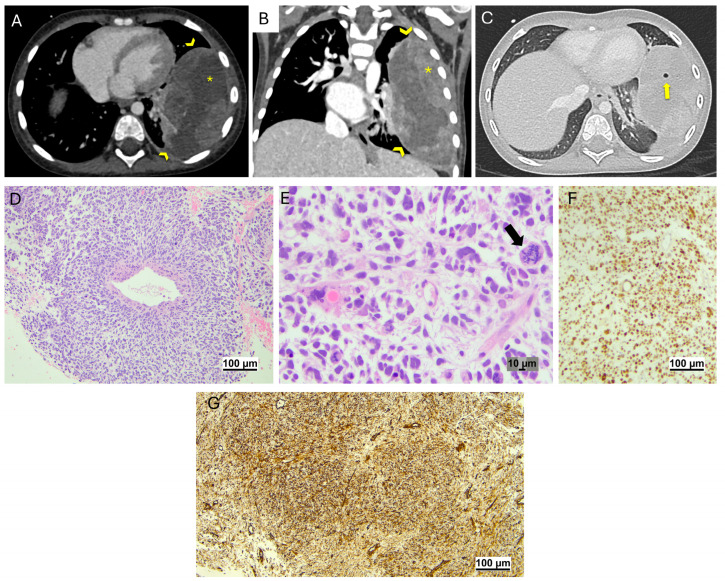
Pleuropulmonary blastoma (*patient 9*). Volumetric chest CT scan with post-CM acquisition, 0.6 mm slice thickness, and 1 mm MPR. Axial scan with mediastinal window (**A**) reveals a big round lesion in the left lung (arrowheads in (**A**)), with inhomogeneous contrast enhancement and liquid (*) and solid areas. Coronal scan with mediastinal window reveals that the lesion (arrowheads in (**B**)), characterized by solid components adjacent to the pleura (*), occupies most of the hemithorax. Axial scan with lung window shows air components within the lesion (arrow in (**C**)). Histopathologic features. Hematoxylin and Eosin: a primitive sarcoma with high cellularity showing a pattern resembling rhabdomyosarcoma (**D**). Note the high-grade malignant cells with hyperchromatic and enlarged nuclei and atypical mitoses (arrow: an atypical mitosis in (**E**)). A strong expression of p53 (**F**) and Vimentin (**G**) was detected by IHC.

A 2-year-old girl (patient 9) was referred to our hospital for persistent fever and dyspnea. A chest X-ray revealed left chest opacification with contralateral midline shift, and a chest CT showed a large solid and cystic mass in the left upper lobe, adherent to the parietal pleura (Figure 6). A thoracoscopy core needle biopsy revealed a neoplasm with high-grade malignant cells, hyperchromatic and enlarged nuclei, and regions of anaplasia. Rhabdomyosarcomatous differentiation was present, and immunohistochemistry confirmed desmin and myoglobin positivity. The differential diagnosis included pleuropulmonary blastoma (PPB) and rhabdomyosarcoma. Genetic analysis confirmed the presence of hotspot mutation in the *DICER1* gene (c.5425G>A) and, in conjunction with the histopathological and radiological findings, supported a type II PPB diagnosis. The patient is receiving neoadjuvant chemotherapy, which includes ifosfamide, vincristine, actinomycin D, and doxorubicin, with partial response at CT evaluations. After the first cycle, she developed Posterior Reversible Encephalopathy Syndrome, now resolved. The next interventions will be surgery and radiotherapy.

## 4. Discussion

Primary lung tumors in pediatric patients are rare and, despite a generally malignant nature, often have a favorable survival prognosis when diagnosed and treated appropriately [9,10]. Delayed diagnoses due to nonspecific symptoms, such as recurrent pneumonia, remain a key issue, as observed in our cohort and previous studies [11]. The male predominance in our series aligns with previous reports on pediatric lung tumors [12,13].

Carcinoid tumors, classified as low-grade neuroendocrine carcinomas, were the most frequently encountered histological type in our series, confirming previous reports that identified them as the most common primary malignant lung neoplasms in children [5]. The mean age at diagnosis was 12.75 years, which again is consistent with the existing literature [13,14]. Delayed diagnosis remains a concern, with patients often presenting with nonspecific respiratory symptoms, leading to misdiagnosis and tumor progression [15,16]. Intrathoracic lymph nodes are the most frequent metastatic site, as observed in patient 4, while diffusion to the liver and skeletal system are rare in pediatric cases [17]. Complete surgical resection remains the cornerstone of treatment, favoring long-term survival. Lymph node dissection is recommended for atypical carcinoids, which have higher malignant potential and risk of metastasis [10]. In our cohort, a patient with atypical carcinoid and lymph node involvement required a pneumonectomy, in line with reports associating lymph node metastases with increased recurrence risk and worse prognosis [18]. The few pediatric data available report a 10-year survival rate of 80–98% for typical carcinoids and a 70–80% range for atypical carcinoids, depending on lymph node involvement and surgical radicality [10,11]. Although the follow-up duration in our study (mean: 51 months, range: 12–84 months) is shorter than the 10-year survival rates reported in the literature, the results are promising, with no recurrences observed during this period. Longer follow-up is needed to fully assess long-term survival rates.

Inflammatory myofibroblastic tumors (IMTs) were identified in two patients, both undergoing successful atypical lobectomy with no recurrence at follow-up. IMT represents the most common mesenchymal lung tumor under the age of 16 [19]. Although traditionally considered benign, recent molecular evidence suggests that a subset of IMTs can exhibit locally aggressive behavior and, in rare cases, distant metastasis [19]. One patient presented with recurrent chest pain, whereas the other was asymptomatic and diagnosed incidentally through a chest CT performed after a thoracic trauma. This aligns with the frequently silent nature of IMT, which is primarily attributed to its peripheral location [20]. Incomplete resections carry a 25% recurrence risk [3,21], but our results confirm that complete surgical resection is associated with excellent long-term prognosis, and reported survival rates exceed 90% [21,22].

Congenital peribronchial myofibroblastic tumor (CPMT) is extremely rare, with less than 30 cases reported [23,24]. The exact pathogenesis remains unclear, the prevalent hypothesis being that it originates from pluripotent mesenchymal cells surrounding proximal bronchial branches during early gestation. After complete surgical resection there was no recurrence, as in previous reports with favorable prognosis following radical excision [24]. Given the benign nature of CPMT, no adjuvant therapy is usually required.

Myoepithelial carcinoma, a very rare pulmonary malignancy, presents unique challenges due to its propensity for local recurrence, as observed in the reported patient. Despite undergoing neoadjuvant chemotherapy, surgical tumor debulking, and adjuvant radiotherapy, local relapse occurred, a not unusual event in consideration of the aggressive potential of tumors > 4 cm and with high mitotic activity [25]. These neoplasms need intensive post-surgical follow-up [26]. No standard treatment protocol exists, but multimodal strategies including radiotherapy and chemotherapy have been used in aggressive cases, with variable success rates [27,28]. Our findings reinforce the necessity of long-term surveillance, particularly in large or high-grade tumors.

Pleuropulmonary blastoma (PPB) is also an aggressive pediatric lung malignancy. Prognosis is worse for Type II and III than Type I [4]. Patient 9 with Type II PPB, was diagnosed by histopathological findings and a *DICER1* mutation that is found in the majority of cases, but it is not clearly correlated with the clinical outcome [29]. The patient is currently receiving neoadjuvant chemotherapy, with further treatment planned, including surgical resection aimed at complete tumor removal, followed by radiotherapy to reduce the risk of recurrence. The literature reports 5-year survival rates of 50–70% for Type II PPB, with outcomes heavily influenced by extent of surgical resection and chemotherapy response [4,29].

Surgical technique remains a pivotal aspect in the management of pediatric lung tumors [16,30,31,32]. The choice between lobectomy, segmentectomy, and sleeve resection depends on tumor location, size, and histology. In our series, lung-sparing procedures were favored whenever technically feasible, in alignment with recent guidelines emphasizing preservation of lung parenchyma to minimize long-term respiratory complications [8,33,34,35]. Of the seven patients treated with surgery, only one required pneumonectomy due to the extent of the disease. Minimally invasive approaches, such as endoscopic resection, are debated as their long-term efficacy remains uncertain [7,36].

Bronchoscopy is crucial for diagnosing and managing endobronchial tumors in children, allowing for the safe acquisition of tissue samples and assisting in bronchial resection during surgery to avoid unnecessary tissue excision [37].

Table 2 summarizes these rare tumors, including their clinical course and treatment strategies.

Consistently with previous research, our findings confirm that surgery remains the cornerstone of treatment, with lung-sparing techniques prioritized when feasible. These cases underscore the importance of complete resection and long-term surveillance, particularly for tumors with a higher risk of recurrence, such as atypical carcinoids and myoepithelial carcinoma. Nodal involvement in atypical carcinoids and larger tumor size in myoepithelial carcinoma are key factors increasing recurrence risk, necessitating extended follow-up. In aggressive cases like PPB Type II/III, a multimodal approach combining chemotherapy, surgery, and radiotherapy improves survival, though the suboptimal outcomes highlight the need for innovative therapeutic strategies.

We recommend prompt evaluation in a tertiary center for pediatric patients with recurrent pneumonia, unexplained respiratory symptoms, refractory asthma, or persistent chest imaging abnormalities such as atelectasis. Early specialist referral and timely chest CT are essential for the accurate and timely diagnosis of primary lung tumors. Individualized treatment plans remain crucial, as no single strategy is suitable for all tumor types.

Our study has several limitations. First, the retrospective design of the study introduces the potential for selection bias and incomplete data, which may affect the generalizability of the findings. The small sample size from a single institution cohort reduces the robustness of the results. In addition, the inclusion of various histological subtypes, each with unique biological characteristics, adds complexity to the interpretation of survival and recurrences. Another key limitation is the follow-up duration, which was not long enough to reliably assess long-term survival, and the data are insufficient to calculate long-term survival rates. Finally, two patients are still under treatment, and their long-term outcomes remain uncertain.

## 5. Conclusions

This study illustrates the wide spectrum of primary lung tumors in pediatric patients and reinforces the requisite for early detection and personalized treatment strategies. The positive outcomes in this cohort emphasize the importance of a comprehensive, multidisciplinary approach to diagnosis and treatment.

Given the rarity of these tumors, future research should prioritize large, multicenter prospective studies to refine treatment protocols and clarify long-term prognostic factors. Additionally, efforts should focus on developing standardized imaging and management guidelines to improve early identification of high-risk cases and facilitate timely interventions.

## Figures and Tables

**Table 1:** Summary of characteristics in primary lung tumor pediatric patients of this study. Legend: F: female; M: male; m: months; N/A: not applicable/not available; y: years.

Patients	Gender	Presenting Symptoms	Chest CT Findings	Method of Diagnosis	Treatment	Outcome and Time to Most Recent Follow-Up	Preexisting Diseases/ Germline Genetics
Diagnosis	Age at Diagnosis	Symptoms Duration		Ki67 Index			
Patient 1	M	Persistent cough and wheezing	A solid lesion with contrast enhancement obstructing the right intermediate bronchus	Endobronchial biopsy	Right lower and middle lobectomy	Alive, disease-free 4 y and 6 m after surgery	N/A
Typical carcinoid tumor	10 y	1 y		1–2%			
Patient 2	M	Recurrent pneumonia	Solid tumor with contrast enhancement within the right intermediate bronchus	Endobronchial biopsy	Right lower and middle lobectomy	Alive, disease-free 4 y and 7 m after surgery	N/A
Typical carcinoid tumor	11 y	3 y		<1%			
Patient 3	M	Recurrent pneumonia	Solid mass with contrast enhancement located in the right intermediate bronchus	Endobronchial biopsy	First, right middle and lower lobectomy; then, right pneumonectomy with lymphadenectomy	Alive, disease-free 1 y after surgery	N/A
Atypical carcinoid tumor	14 y	6 m		10%			
Patient 4	M	Recurrent pneumonia	Bronchial filling defect in the right lower lobe, causing collapse of the distal parenchyma	Endobronchial biopsy	Right lower lobectomy	Alive, disease-free 7 y after surgery	N/A
Atypical carcinoid tumor	16 y	2 y 2 m		3%			
Patient 5	M	Recurrent chest pain	Mass in the left lower lobe with homogeneous low-contrast enhancement	Thoracoscopic biopsy	Atypical left lower lobectomy	Alive, disease-free 5 y after surgery	N/A
Inflammatory myofibroblastic tumor	9 y	6 m		1–2%			
Patient 6	M	Incidental diagnosis following chest trauma	Mass in the left upper lobe with heterogeneous enhancement	Surgical exeresis	Atypical resection of the left upper lobe	Alive, disease-free 8 y after surgery	Neonatal asphyxia
Inflammatory myofibroblastic tumor	11 y	NA		1–2%			
Patient 7	F	Recurrent right upper lobe pneumonia	Endobronchial lesion obstructing the right upper bronchus	Endobronchial biopsy	Right upper lobectomy	Alive, disease-free 6 m after surgery	Infantile hemangioma on the face
Congenital peribronchial myofibroblastic tumor	5 y	1 y		10%			
Patient 8	F	Persistent cough and dyspnea	Polylobed round lesion of the left lower lobe exhibiting heterogeneous contrast enhancement with a hyperdense halo	Thoracoscopic biopsy	Tumor debulking, adjuvant chemotherapy, and radiotherapy	Alive, stable disease 1 y and 6 m after diagnosis. Ongoing adiuvant chemotherapy	Compound somatic heterozygous mutation in *DICER1* (c.5566G>A and c.4390_4391dup) on tumor tissue
Myoepithelial carcinoma	16 m	3 m		80%			
Patient 9	F	Persistent fever and dyspnea	A large solid and cystic mass in the left upper lobe attached to the parietal pleura	Thoracoscopic biopsy	Neoadjuvant chemotherapy	Alive 6 m after diagnosis. Ongoing neoadjuvant chemotherapy	Somatic *DICER1* mutation (c.5425G>A) on tumor tissue
Pleuropulmonary blastoma type II	2 y	1 m		90%			

**Table 2:** Overview of pediatric lung tumors observed in our study according to the literature.

Tumor Type	Epidemiology	Tumor Behavior and Prognosis	Management
Typical Carcinoid Tumor	~1 per 100.000 individuals, 1% of all lung cancers [38,39]. Most common primary pulmonary tumor in childhood [39]	Malignant (low grade). Slow-growing with a generally favorable prognosis. Minimal risk of metastasis; good long-term survival after surgical resection	Surgery (lobectomy, segmentectomy, or wedge resection for small, peripheral tumors) is the primary treatment. Adjuvant therapy (chemo/radiotherapy) is not typically required
Atypical Carcinoid Tumor	10–15% of pulmonary carcinoids [39]	Malignant (intermediate grade). More aggressive than typical carcinoids, with a higher risk of metastasis, particularly to intrathoracic lymph nodes. Prognosis depends on the extent of resection and metastatic spread	Surgery (lobectomy with mediastinal lymph node dissection) is preferred. Adjuvant chemotherapy (cisplatin and etoposide) is recommended for stage III disease. Radiotherapy may be considered for unresectable cases. Somatostatin analogs, mTOR inhibitors, and anti-VEGF agents may be used in metastatic cases
Inflammatory Myofibroblastic Tumor	0.04–0.7% of all lung neoplasms [19]. The most prevalent primary lung tumor under the age of 16 [21]	Benign to intermediate. Indolent, often asymptomatic or associated with mild symptoms (e.g., chest pain). Risk of local recurrence if not fully excised, but generally good prognosis after complete resection	Surgical excision is the treatment of choice. Chemotherapy is reserved for unresectable cases or incomplete resections. Radiation therapy may provide palliative benefits but is not routinely used after complete resection
Congenital Peribronchial Myofibroblastic Tumor	Extremely rare (~25 cases), typically occurs in utero or infancy [24]	Typically benign with a favorable clinical course post-surgical resection. Long-term follow-up is recommended due to the tumor‘s rapid growth potential	Radical surgery is recommended. No chemotherapy or radiotherapy is usually required. No recurrence or metastasis in cases with complete resection
Myoepithelial Carcinoma	42 case reports documented [28]	Low-grade malignant neoplasm with the potential for local recurrence and distant metastasis. Tumors larger than 4 cm and those with brisk mitotic activity have a higher risk of recurrence. Complete surgical excision correlates with better prognosis	Surgery (lobectomy for small, well-defined tumors) is the preferred treatment. Radiotherapy is recommended for tumors ≥ 4 cm or with positive surgical margins. Chemotherapy (cisplatin, fluorouracil, docetaxel) is considered for metastatic cases but has limited evidence. Targeted therapy (MEK and mTOR inhibitors) is investigational
Pleuropulmonary Blastoma	0.5–1% of all primary malignant lung tumors, primarily in pediatric patients [29]	Aggressive pediatric malignancy classified into Type I (cystic, better prognosis), Type II (mixed cystic/solid), and Type III (solid, poorest prognosis). Type II and III have a higher metastatic potential, with common sites including the lungs, brain, and bones. Long-term follow-up is critical due to recurrence risk	Surgery is the mainstay of treatment. Neoadjuvant chemotherapy may be used in Type II and III before surgery. Adjuvant chemotherapy (vincristine, actinomycin D, cyclophosphamide, doxorubicin) is essential for Type II and III cases. Radiotherapy is considered in cases with incomplete resection or metastatic disease. Genetic testing for *DICER1* mutations is recommended

## Data Availability

The data presented in this study are available on request from the corresponding author.
